# Motivating role of type H vessels in bone regeneration

**DOI:** 10.1111/cpr.12874

**Published:** 2020-07-19

**Authors:** Jiankang Zhang, Jian Pan, Wei Jing

**Affiliations:** ^1^ State Key Laboratory of Oral Diseases National Clinical Research Center for Oral Diseases Department of Oral and Maxillofacial Surgery West China Hospital of Stomatology Sichuan University Chengdu China

**Keywords:** angiogenesis, coupling, micro‐vessels, osteogenesis, type H endothelium

## Abstract

Coupling between angiogenesis and osteogenesis has an important role in both normal bone injury repair and successful application of tissue‐engineered bone for bone defect repair. Type H blood vessels are specialized microvascular components that are closely related to the speed of bone healing. Interactions between type H endothelial cells and osteoblasts, and high expression of CD31 and EMCN render the environment surrounding these blood vessels rich in factors conducive to osteogenesis and promote the coupling of angiogenesis and osteogenesis. Type H vessels are mainly distributed in the metaphysis of bone and densely surrounded by Runx2^+^ and Osterix^+^ osteoprogenitors. Several other factors, including hypoxia‐inducible factor‐1α, Notch, platelet‐derived growth factor type BB, and slit guidance ligand 3 are involved in the coupling of type H vessel formation and osteogenesis. In this review, we summarize the identification and distribution of type H vessels and describe the mechanism for type H vessel‐mediated modulation of osteogenesis. Type H vessels provide new insights for detection of the molecular and cellular mechanisms that underlie the crosstalk between angiogenesis and osteogenesis. As a result, more feasible therapeutic approaches for treatment of bone defects by targeting type H vessels may be applied in the future.

## Graphical abstract

Type H vessels are mainly distributed in the metaphyseal region and sub‐periosteum and show strong positive staining for CD31 and EMCN. They are specialized microvascular components and closely related to bone healing speed through the crosstalk between angiogenesis and osteogenesis.

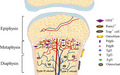

## 1 INTRODUCTION

Large bone defects caused by trauma and disease fail to heal spontaneously and require implantation of biomaterial‐based bone substitutes to achieve bone tissue regeneration. Tissue‐engineered bone provides an option worthy of further investigation for the treatment of large bone defects. Although application of tissue‐engineered bone for treatment of bone defects has made great progress, it still faces the obstacle of slow or absent vascularization.^1^ As a result, it is difficult to achieve an effective vascular network in tissue‐engineered bone within a short time frame, leading to death of seeded cells through a lack of nutrients and oxygen and excessive accumulation of metabolites.^2^ Bone is composed of highly vascularized and innervated tissue.^3^ Blood vessels in bone tissue play important roles in bone growth, development, shaping, remodelling and injury repair. Vascular endothelial cells (ECs) are a component of bone tissue that become involved in all of these processes by interacting with bone cells. These cells have a variety of functions, such as maintaining vascular integrity, promoting bone formation and influencing dynamic balance of osteogenesis and osteoclastogenesis, through various signalling pathways. For both normal bone injury repair and successful application of tissue‐engineered bone for bone defect repair, timely and abundant angiogenesis is essential.^4^

Bone has a unique regenerative capacity after injury that differs from the process in other tissues.^5^ Specifically, the bone regeneration process repeats all of the stages involved in bone development to regenerate new bone, rather than developing scar tissue, and thus restore the original physiological and mechanical properties.^6^ Bone regeneration comprises a series of complex and continuous physiological processes that can be divided into four stages: haematoma formation, fibrous callus formation, bone callus formation and bone shaping plus remodelling.^7^ Numerous studies have shown that inflammatory responses and neovascularization are key factors for initiation of bone regeneration.^8^, ^9^, ^10^ Hausman et al^11^ demonstrated that application of angiogenesis inhibitors to a rat model of femoral fracture inhibited fracture healing and led to atrophic nonunion. Holstein^12^ found that use of the immunosuppressive drug rapamycin, which has anti‐angiogenic properties, inhibited neovascularization in the fracture callus and delayed fracture healing. Vessels can not only maintain the high metabolic demand for nutrients and oxygen in the callus, but also provide pathways for cells such as inflammatory cells, fibroblasts and osteoblast/osteoclast precursors to enter the defect area.

Many studies have shown that osteoblasts, osteoclasts and vascular ECs are linked by signalling molecules that promote another.^13^, ^14^, ^15^ As a consequence, the intimate spatial and temporal link between osteogenesis and angiogenesis has been termed “angiogenic‐osteogenic coupling.”^16^ Two subtypes of vascular ECs with completely different morphological, molecular and functional characteristics have been identified in developing bone tissues, namely type H vascular ECs with high expression of CD31 and EMCN, and type L vascular ECs with low expression of CD31 and EMCN. Type H vascular ECs are specifically distributed in the metaphyseal region, and the numbers of type H blood vessels and surrounding osteogenic progenitor cells decrease significantly with increasing age.^13^

In this article, we review the motivating role of type H vessels in osteogenesis and provide a summary of feasible therapeutic approaches targeting type H vessels for the regeneration of bone defects.

## 2 GENERAL CHARACTERISTICS OF TYPE H VESSELS

**Figure 1:**
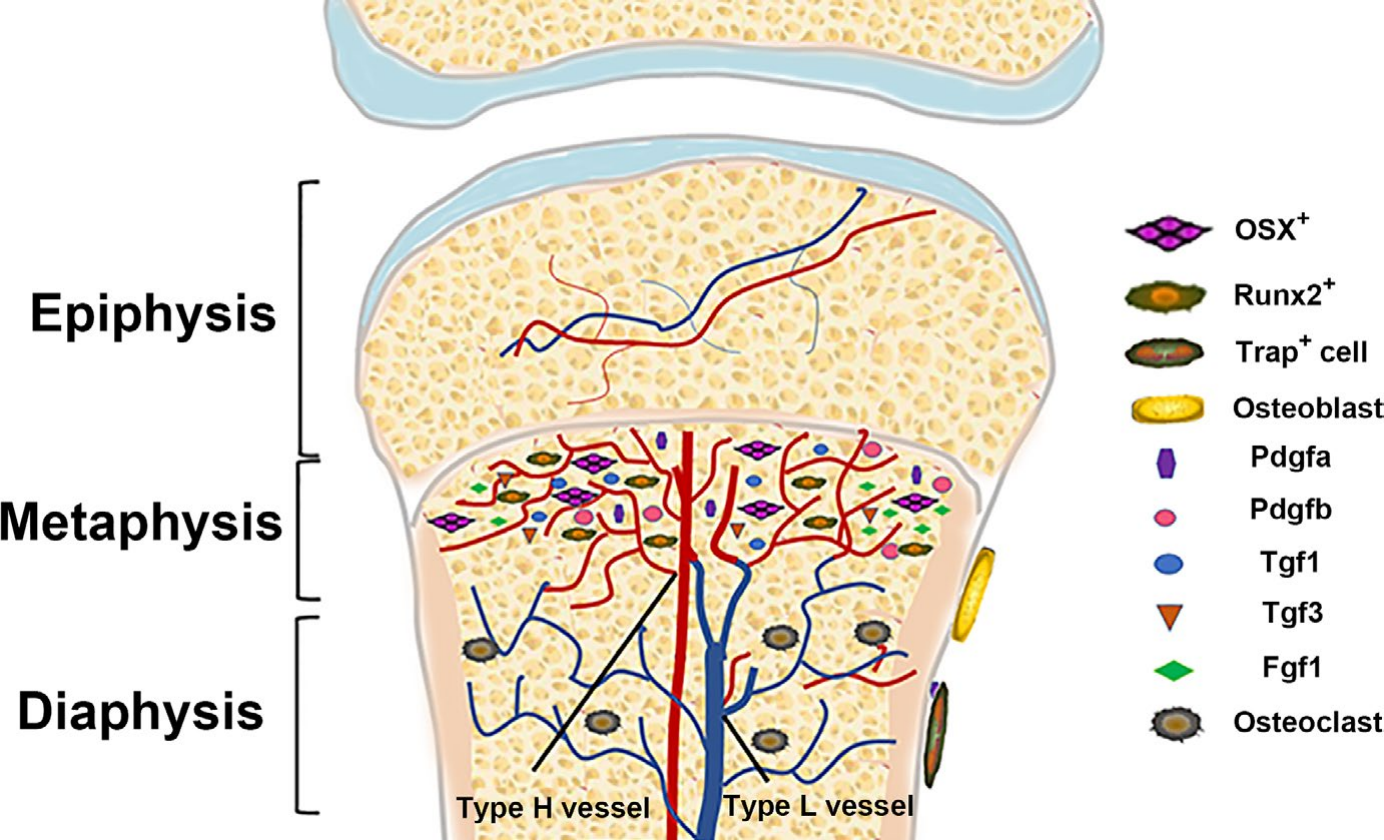
General characteristics of type H vessels. Type H vessels are mainly distributed in the metaphyseal region and sub‐periosteum and show strong positive staining for antibodies against two kinds of EC proteins (CD31 and EMCN), while type L vessels are distributed in the diaphyseal region and show weak positive staining for CD31 and EMCN. These two types of vessels are closely connected at the epiphysis‐diaphysis junction to form a complete vascular bed. Secretion of Pdgfa, Pdgfb, Tgf1, Tgf3 and Fgf1 from type H ECs is significantly higher than that from type L ECs. More Runx2^+^ and Osx^+^ osteoprogenitors surround type H vessels

There are two subtypes of vessels, type H vessels and type L vessels, in the capillaries of the metaphysis and bone marrow cavity (Figure 1). These vessels are classified according to their specialized ECs that have specific molecular and morphological properties.^17^ Type H vessels are mainly distributed in the metaphyseal region and sub‐periosteum and show strong positive staining with antibodies against two kinds of EC proteins (CD31 and EMCN), while type L vessels are mainly distributed in the diaphyseal region and show weak positive staining for CD31 or EMCN.^11^, ^15^, ^18^ Type H vessels are interconnected by distal vessel loops or arches and resemble straight columns, while type L vessels mainly located in the diaphysis display a highly branched pattern characteristic of the sinusoidal vasculature of the bone marrow. These two types of vessels are closely connected at the epiphysis‐diaphysis junction and form a complete vascular bed in the bone marrow cavity.^13^

Although type H ECs only account for 1.77% of all bone ECs and 0.015% of total bone marrow ECs, a large number of bone progenitor cells, which can differentiate into osteoblasts and osteocytes, are distributed around these vessels, suggesting that type H vessels may be a potent promoter of bone regeneration.^19^ The dense distribution of Runx2^+^ osteoprogenitors and osteoblasts around these CD31^+^ vessels in the metaphysis and endosteum confirms their role as a potent promoter of bone regeneration.^20^ In contrast, type L vessels have almost no surrounding bone progenitor cells.^13^, ^17^ The two types of ECs were isolated and purified from bone tissue and evaluated for their levels of mRNA expression for secreted growth factors that promote the survival and proliferation of bone progenitor cells. The transcript levels for Pdgfa, Pdgfb, Tgbf1, Tgfb3 and Fgf1 in type H ECs were significantly higher than those in type L ECs. These secretory growth factors are closely related to the proliferation and survival of bone progenitor cells.^13^ Thus, the findings further confirm a key role for type H vessels in bone regeneration. Langen et al^18^ showed that type H vessels can transit to type L vessels, suggesting that type H ECs may be the upstream ECs in bone.

It is well known that the number of osteoblasts in aged individuals is much lower than that in young individuals.^21^ This change also applies to type H vessels. Wang et al^22^ demonstrated that the number of type H vessels was significantly decreased in not only aged mice, but also ovariectomized mice, a widely used model of postmenopausal osteoporosis. Similar results were found in aged and osteoporotic individuals, suggesting the existence of type H vessels in human bones and their use as a sensitive marker for ageing and bone mass.^23^ In fact, the proliferation capacity of vascular ECs in bone marrow was mainly determined by type H vascular ECs. The proliferation ability of type H vascular ECs in young individuals was significantly stronger than that in adult individuals, while the proliferation ability of type L vascular ECs did not differ significantly between young and old individuals. Yan et al isolated type H ECs using microbeads and demonstrated high expression of Notch signalling in these cells. Furthermore, blockade of Notch signalling using DAPT decreased not only the Notch signalling, but also CD31 and EMCN expression.^24^ Some researchers treated human umbilical vein endothelial cells (HUVECs) with ophiopogonin D and stimulated CD31 and EMCN expression. CD31^+^ and EMCN^+^ HUVECs had stronger migration and vascularization capacity than other types of vascular ECs in vitro and in vivo.^25^

## 3 ROLE OF TYPE H VESSELS IN BONE REGENERATION

Bone regeneration comprises a series of complex and continuous physiological processes that can be divided into four stages: haematoma formation, fibrous callus formation, bone callus formation and bone shaping plus remodelling.^7^ And the process is regulated by complicated interactions between the numerous cell types found in bone, mainly BMSCs, osteoblasts, osteoclasts and osteocytes. BMSCs, located in the bone marrow, are the precursor cells of osteoblasts. They differentiate into osteoblasts and migrate to bone surface to take part in bone regeneration. osteoclasts, as we know, play a crucial role in bone formation and resorption. In recent years, numerous strategies have been detected to accelerate the process of tissue engineering and regenerative medicine fields, such as bone bioactive material,^26^, ^27^ extracellular vesicles (EVs) strategy^28^ and stem cell transplantation therapy.^29^ Among the promising therapies, vascularization is one of the problems that cannot be ignored. The bioactive scaffold used in bone tissue engineering should be biocompatible and allow blood vessels colonization. In addition, scaffold enriched EVs, that is associated with an enhanced vascularization providing a novel regulatory system in osteo‐angiogenesis evolution.^30^, ^31^

During bone development and bone regeneration, the migration of osteogenic precursor cells to the bone defect area is tightly related to the invasion of blood vessels. During defect repair, osteogenic precursor cells and blood vessels invade the bone defect area together to promote the formation of new bone.^32^ Blood vessels not only form a local circulation to obtain nutrients and oxygen in the newly formed bone area, but also directly promote bone formation. The endovascular blood sinus is the initial site of osteogenesis, and sufficient nutrients, oxygen and minerals directly promote the formation and mineralization of an osteogenic matrix.^33^, ^34^ Type H vessels have a higher tendency to differentiate into arteries than type L vessels, suggesting a close relationship between type H vessels and newly formed vessels, especially new formed arteries.^18^ The formation of new arteries is the basis of local vascular network formation in bone tissue, which is crucial for bone regeneration in a bone defect area.

The osteogenic progenitor cells surrounding type H vessels express the transcription factors Osterix and Runx2, which promote bone formation. Kusumbe et al^13^ found that although type H ECs comprise less than 2% of ECs, more than 82% of Runx2^+^ and 70% of Osterix^+^ osteoprogenitors are selectively positioned around the type H endothelium. Furthermore, type H ECs secrete many factors that stimulate the proliferation and differentiation of osteoprogenitors to regulate osteogenesis. Ramasamy et al demonstrated that Notch signalling in type H vascular ECs plays a key role in mediating vascular formation and osteogenic coupling. Cell proliferation and high expression of Noggin protein occur in type H vascular ECs under the action of Notch signalling. Noggin protein can promote proliferation and differentiation of osteoblastic progenitor cells as well as maturation and hypertrophy of chondrocytes. Mature and hypertrophic chondrocytes can guide vascular budding through high secretion of VEGF and promote the generation of new blood vessels. Through the combination of Notch signalling by ECs, Noggin and VEGF, the processes of osteogenesis and angiogenesis are linked together.^35^ After blockade of Notch signalling in ECs, vessel disorganization, cartilage defects, reduced bone trabeculae and many other low osteogenesis manifestations were found in bone marrow. These results indicate that type H vessels play important roles in mediating growth of blood vessels, maintaining number of perivascular bone progenitor cells and coupling of osteogenesis and angiogenesis (Table 1).

**Table 1:** The stimulating roles type H vessels play are summarized below

**Stimulating factor**	**Mechanism**	**References**
Dense arrangement of osteoprogenitors	Osterix^+^, Runx2^+^ and collagen type 1α^+^ osteoblast cells are densely arranged around type H vessel, which give rise to osteoblasts and osteocytes	13, 17, 20
Growth factors secreted by type H vessel ECs	Type H vessels can promote bone formation by secreting various factors that stimulate proliferation and differentiation of osteoprogenitors in the bone marrow	13, 35
New arteries formation	Type H vessels show a closer relationship to newly formed vessels and have a higher tendency to differentiate into arteries than type L vessels	18
Initiation condition for osteogenesis	The newly formed artery network is the basis of local vascular network formation and provides necessary nutrients to meet the metabolic demands of osteogenesis in a bone defect area	18
Matrix metalloproteinase‐9 (MMP‐9)	MMP‐9 released from type H ECs, not osteoclasts, are essential for resorbing cartilage to lead longitudinal bone growth	87

## 4 FACTORS INVOLVED IN BONE REGENERATION BY REGULATING TYPE H VESSEL FORMATION

**Figure 2:**
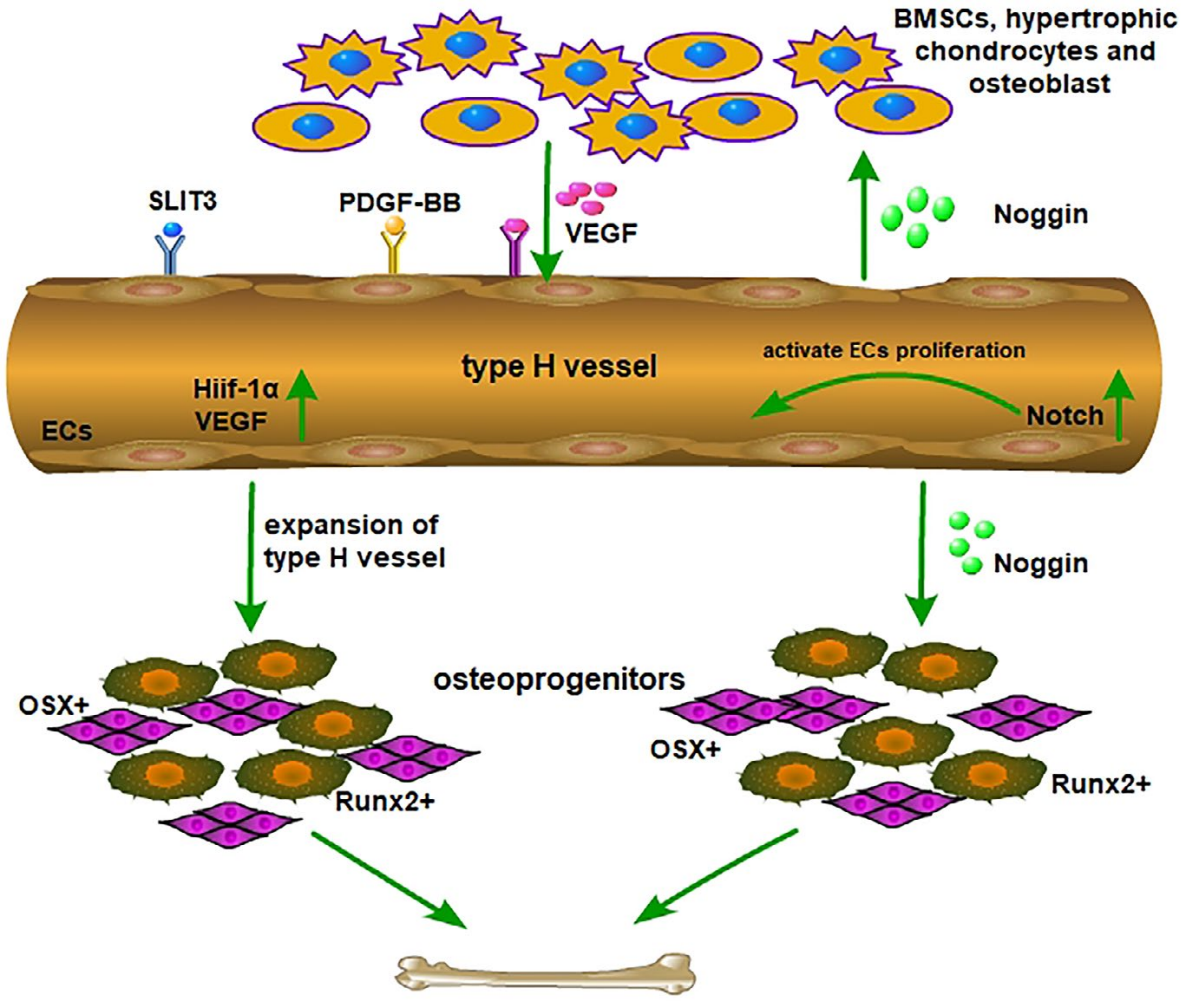
Multiple signalling factors are involved in coupling of type H vessel formation and osteogenesis in the bone marrow microenvironment. Multiple cell lineages, including hypertrophic chondrocytes, osteoblast lineage cells, vascular ECs and osteoclasts promote type H vessel formation by secreting VEGF, SLIT3 and PDGF‐BB. RANKL secreted by type H vascular ECs supports vessel‐associated osteoclasts through a RANKL‐RANK signalling mechanism to facilitate bone regeneration

**Figure 3:**
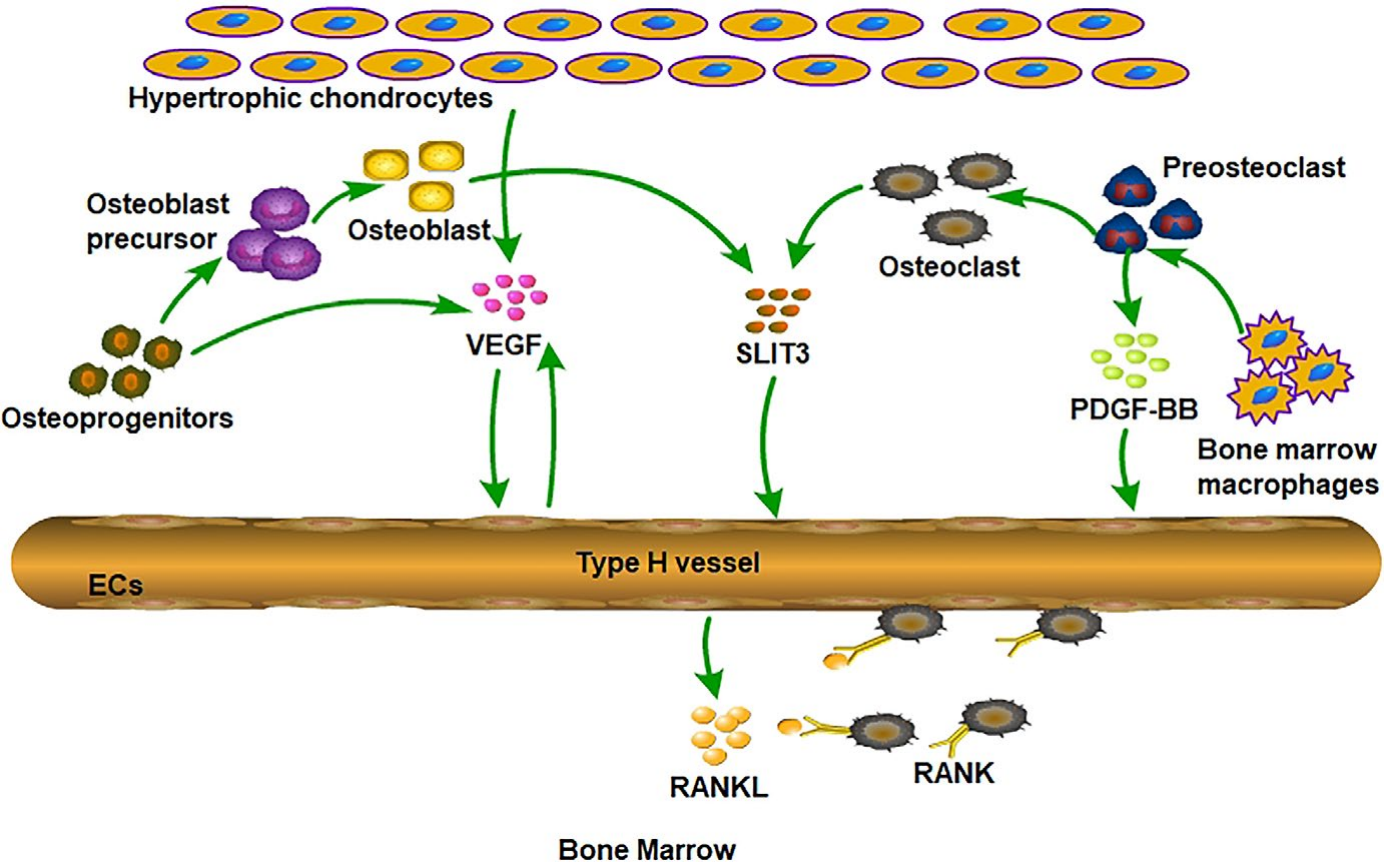
HIF‐1α and Notch signalling participate in regulation of coupling for type H vessel formation and osteogenesis. High levels of Hif‐1α expression in cells lead to pronounced expansion of type H endothelium and Runx2^+^ and Osterix^+^ osteoprogenitors. Type H vascular ECs show strong cell proliferation capacity and high Noggin protein expression under the action of Notch signalling. In addition, Noggin protein promotes the proliferation and differentiation of osteoblasts and the maturation and hypertrophy of chondrocytes. Mature and hypertrophic chondrocytes guide vascular sprouting and promote the formation of new blood vessels

Various types of cells in bone marrow influence cell proliferation and differentiation through direct or indirect cellular connections and by secreting exocrine factors. To date, a variety of signalling molecules related to vasculogenesis, osteogenesis and osteoclasts have been shown to be involved in the angiogenesis‐osteoclast coupling process. Preosteoclasts and osteoblasts secrete factors such as platelet‐derived growth factor type BB (PDGF‐BB) and slit guidance ligand 3 (SLIT3) to induce the migration, proliferation and differentiation of bone mesenchymal stem cells and ECs^17^, ^36^ (Figure 2). Many signalling pathways, such as the HIF‐1α and Notch pathways, participate in the regulation of vessel assembly and bone regeneration (Figure 3).

### 4.1 PDGF‐BB promotes type H vessel formation and osteogenesis by stimulating cell migration and differentiation

PDGF‐BB is a dimeric cationic glycoprotein that is mainly secreted by preosteoclasts, the immature precursors of resorptive osteoclasts, in bone marrow. The receptors for PDGF‐BB are cell membrane tyrosine kinases known as platelet‐derived growth factor receptor (PDGFR)‐α and PDGFR‐β.^37^ Xie et al showed that PDGF‐BB can induce type H vessel formation during bone formation. In addition, bone mass and type H vessels were significantly decreased when endogenous PDGF‐BB was blocked in mice.^36^

Studies have shown that PDGF‐BB regulates mesenchymal stem cell migration, differentiation and mineralization through binding to PDGFR‐β and triggers the mitogen‐activated kinase and phosphoinositide‐3 kinase/Akt signalling pathways.^38^, ^39^ When a bone defect occurs, monocyte/macrophage lineage cells such as tartrate‐resistant acid phosphatase‐positive (TRAP^+^) mononuclear cells and osteoblastic cells move to the bone defect area and secrete PDGF‐BB to recruit PDCs for osteogenesis. In addition, PDGF‐BB secreted by preosteoclasts induces formation of the CD31^hi^Emcn^hi^ vessel subtype to promote the coupling of type H vessels with bone formation.^17^ When the expression level of PDGF‐BB was knocked down in adult mice, the number of type H vessels was significantly decreased, accompanied by reductions in trabecular bone and cortical bone mass. Treatment of osteoporosis model mice with Cathepsin K inhibitors to increase the number of osteoclast precursor cells or exogenous PDGF‐BB reversed the decrease in type H vessels and promoted bone regeneration. Meanwhile, inhibition of preosteoclast differentiation into osteoclasts and increased concentration of bone marrow PDGF‐BB in osteoporosis model mice promoted the formation of type H vessels.^40^, ^41^

PDGF‐BB was shown to promote osteogenesis through stimulation of osteoprotegerin (OPG), a major regulator of osteoclastogenesis, bone resorption and vascular calcification, production in osteoblastic cells. PDGF‐BB released from platelets at a fracture site may be responsible for the initial rise in OPG production by surrounding stromal cells and pre‐osteoblasts.^42^ Furthermore, stimulated OPG production was beneficial for growth of type H vessels and bone formation at the fracture site.^43^, ^44^ Another role for PDGF‐BB at the site of a bone defect was the production of OPG to enhance local angiogenesis and/or preserve bone in the short term that may otherwise be lost during the wound healing and remodelling processes.^42^

### 4.2 SLIT3‐ROBO activation promotes vascular network formation and bone regeneration

Slit guidance ligand 3 was first discovered in *Drosophila* by Niisslein‐Volhard et al^45^ in 1984 as a secreted extracellular matrix protein expressed and secreted by glial cells in the midline of the nervous system, and was considered an axon‐guiding molecule. SLIT3‐like proteins, such as SLIT1 and SLIT2 proteins, are highly conserved in structure, including an N‐terminal signal peptide that guides protein synthesis and four leucine‐rich repeating units (d1‐d4), together with 9 (in mammals) or 7 (in non‐mammals) epidermal growth factor repeats, one laminin‐G‐like domain, and one C‐terminal cysteine domain.^46^ Slit3 is located in mitochondria and widely distributed in the brain, heart, lung, kidney, skeletal muscle, tongue, spleen, lymph node, diaphragm, umbilical vein and other tissues.^47^, ^48^ It is also expressed in vascular ECs and smooth muscle cells.^49^ In a bone defect area, osteoblast‐derived Slit3 binds to Robo1 receptor and activates Rac1 through activation of intracellular Rho GTPase, triggering intracellular actin cytoskeletal rearrangement and promoting proliferation and migration of HBVSMCs.^50^ Zhang et al^51^ further showed that Slit3 is the functional ligand of Robo4 and that Slit3 interacts with Robo4 to induce angiogenesis.

Osteoblast‐derived SLIT3 enhances an osteogenic subtype of vascular endothelium, CD31^hi^EMCN^hi^ endothelium, in bone. Mice lacking SLIT3 or the known SLIT receptor ROBO1 displayed reductions in both bone marrow CD31^hi^EMCN^hi^ endothelium and basal bone mass. Proof of principle that exogenous SLIT3 promoted bone defect healing and prevented bone loss in a postmenopausal osteoporosis model was also provided.^17^ Meanwhile, ROBO1‐deficient mice showed an osteopenic phenotype, and knockdown of ROBO1 partially blocked the response of bone marrow ECs to SLIT3 stimulation.^52^, ^53^ However, the possible contribution of other SLIT‐ROBO members to this regulation process could not be excluded.^54^ Osteoclast‐secreted SLIT3 regulates not only the bone resorption process, but also the bone regeneration process. Elimination of Slit3 or its receptor, Robo1, in mice resulted in osteopenic phenotypes due to decreased bone formation and increased bone resorption. Furthermore, mice deficient in Slit3 or its receptor Robo1 exhibited significantly reduced type H endothelium. These results suggest that Slit3 takes part in the angiogenesis process and also affects the bone formation process.^55^

### 4.3 Endothelial Hif‐1α regulates angiogenesis and osteogenesis

Hif‐1 has been identified as a transcriptional activator of erythropoietin (EPO), a core molecule in the mechanism for adaptation to oxygen changes.^56^ Hif‐1 mainly exists as a heterodimer of Hif‐1α and Hif‐1β subunits.^57^ HIF‐1β is an aryl hydrocarbon receptor nuclear translocator, and its expression and oxygen connection are not strong. However, the Hif‐1 gene is sensitive to oxygen^58^ and is located on human chromosome 14q21‐q24.^59^ Von Hippel‐Lindau (VHL) binds to hydroxylated Hif‐1α and subsequently degrades under the action of Fe^2+^ and oxygen, while inactivation of VHL under hypoxic conditions prevents it from binding to hydroxylated Hif‐1α, thus affecting the proteasomal degradation of Hif‐1α. When the dynamic balance between synthesis and degradation of Hif‐1α was disrupted, Hif‐1α was overexpressed in cells.^60^

Hif‐1α can induce VEGF expression in hypoxic or ischaemic cells,^61^ and VEGF has an important role in angiogenesis and vascular remodelling.^62^ HIF‐1α in ECs significantly promotes the formation of type H vessels in the metaphysis. Through its role in up‐regulating VEGF‐A expression in hypoxic tissues, the activity of HIF‐1α in these tissues plays a key role in wound healing and tumour angiogenesis in mice.^63^ EC‐specific deletion of the Vhl gene in mice led to pronounced expansion of type H endothelium and metaphyseal vessel columns, and finally resulted in metaphysis expansion, increased formation of trabecular bone, and higher numbers of Runx2^+^ and Osterix^+^ osteoprogenitors in the mutant mice. Meanwhile, loss of type L ECs and expansion of CD31^hi^Emcn^hi^ endothelium after irradiation significantly promoted HIF‐1α expression at both the protein and gene transcript levels. Above all, HIF‐1α plays a key role for induction of type H ECs.^17^ When a bone defect or fracture occurs, local vascular damage leads to haematoma formation. The haematoma and edge of the injured area are in a state of low oxygen tension (<1%).^64^ Hypoxic stimulation induces an increase in transcription of Hif‐1α, an important regulator of angiogenesis under physiological or pathological conditions. The high level of Hif‐1α expression in cells, including ECs, BMSCs and osteoblasts, in the bone defect area leads to pronounced expansion of type H endothelium and metaphyseal vessel columns. Finally, metaphysis expansion, increased formation of trabecular bone, and higher numbers of Runx2^+^ and Osterix^+^ osteoprogenitors can be found in the bone defect area.^13^, ^63^, ^64^ Kusumbe et al^17^ showed that Hif‐1α expression was very high in type H vascular ECs isolated from the long bones of mice. Application of gene mutation technology to reduce the protein expression of Hif‐1α caused a significant increase in the number of type H blood vessels in the long bones of mice, as well as the number of epiphyseal trabeculae and osteogenic (progenitor) cells, indicating an important role of Hif signalling in the regulation of osteogenesis and angiogenesis. Increased expression of Hif‐1α can directly induce transcription and translation of the VEGF gene.^65^ Many reports have confirmed important roles for VEGF in promoting angiogenesis and osteogenesis.^66^, ^67^, ^68^

### 4.4 Notch signalling regulates type H vessel formation

The Notch signalling pathway is involved in cell proliferation, differentiation and apoptosis.^69^ In mammals, the Notch signalling pathway contains four Notch receptors, Notch1‐4 and five ligands, Delta‐like (DLL) 1, DLL3, DLL4, Jagged1 and Jagged2.^70^ Notch receptors are transmembrane proteins composed of a Notch intracellular domain, transmembrane domain and Notch extracellular domain.^71^, ^72^ The Notch signalling pathway is closely related to bone microenvironment angiogenesis, which can promote the proliferation and angiogenesis of vascular ECs in the long bones of mice.^13^ Ramasamy reported that increasing notch signalling via genetic operation of a notch receptor inactivator (*Fbwx*) led to type H vessels, Runx2^+^ osteoprogenitors, and EC Noggin secretion increase. In addition, inhibition of notch signalling by diminishing DLL4 resulted in a decrease of type Noggin expression, type H vessels and EC proliferation. Osteoblastic differentiation, bone formation and endochondral ossification were also blocked when notch signalling was inhibited.^35^ Type H ECs show strong cell proliferation capacity and high Noggin protein expression under the action of Notch signals. In addition, Noggin protein can promote the proliferation and differentiation of osteoblasts and the maturation and hypertrophy of chondrocytes. Mature and hypertrophic chondrocytes guide vascular sprouting and promote the formation of new blood vessels by secreting VEGF (Figure 3). The combination of Notch signalling, Noggin, and VEGF links the osteogenesis and angiogenesis processes. Meanwhile, the normal vascular structure in bone exhibited disordered morphology, cartilage defects, shortening of long bones, reduced number of trabeculae and loss of bone mass when Notch signalling in ECs was blocked. These results indicate that the H‐shaped blood vessels existing in bone tissue play important roles in mediating growth of blood vessels, maintaining numbers of bone progenitor cells around blood vessels and coupling of osteogenesis and angiogenesis. Furthermore, bone angiogenesis declines in elderly mice through inhibition of the Notch signalling pathway, while activation of the Notch signalling pathway can reverse the decline in bone angiogenesis caused by ageing, restore the normal bone formation rate in mice and maintain the normal bone mass and bone density in the body.^20^, ^73^

Even though angiogenesis and EC differentiation are inhibited by activation of the Notch signalling pathway in some tissues, such as embryo, mammalian retina and tumour tissues, Notch in ECs has the opposite role when it comes to bone tissue.^74^ Despite the finding that excessive activation of the Notch signalling pathway led to a significant decrease in retinal blood vessels,^75^ Ramasamy et al^76^ demonstrated that the total numbers of ECs, type H vessels, and arterioles were remarkably increased at the metaphysis of long bones. Moreover, the expression level of flow‐modulated genes, including Klf2, Nos3 and Pecam1, was upregulated in ECs through activation of the Notch signalling pathway in bone tissues.^76^

The Notch signalling pathway can also coordinate with HIF‐1α to participate in the regulation of type H capillary generation, increasing type H capillaries and bone progenitor cells to regulate bone formation under hypoxic conditions.^77^ HIF‐1α levels increase under hypoxia stress, and its activity is regulated by prolyl hydroxylase domain protein (PHD). Under normoxic conditions, targeted HIF‐1α can be degraded by PHD with oxygen, meaning that HIF‐1α can maintain a more stable state under hypoxic conditions.^78^ HIF‐1α signalling induces expression of VEGF and stimulates initiation of angiogenesis through paracrine signal transduction in vascular ECs, coordinating the Notch signalling pathway to regulate the generation of type H capillaries.^79^ The overexpression or blockage of miR‐497B195 cluster regulated endogenous level protein of Fbxw7 and P4HTM.^80^ Fbxw7 mediates active notch polyubiquitination and proteasomal degradation whereas P4HTM regulates Hif‐1α degradation.^81^, ^82^ Murine treated by miR‐195 depletion led to an increase of type H endothelial cells, as well as an increase of osteoblasts and Osterix^+^ osteoprogenitors on bone surface. And the volume of trabecular bone and the thickness of trabecular bone increased while the trabecular separation decreased.^80^

### 4.5 Other potential regulatory factors

Some studies have shown that low‐intensity pulsed ultrasound (LIPUS) can promote blood flow and angiogenesis in the fracture healing process.^83^, ^84^ Xu et al^85^ found that LIPUS induced substantial increases in osteoblast proliferation and type H vessel number in a rat spinal fusion model. The matrix modification properties of matrix metalloproteinases (MMPs) can also affect angiogenesis and bone formation.^86^ MMP‐2 promoted a marked increase in the vessel number in bone,^87^ while type H ECs released MMP‐9 with a key role in resorption of articular cartilage during growth of long bones.^88^

## 5 FUTURE INSIGHTS AND CONTROVERSIAL ISSUES

Multiple cell activities and orchestration of various signalling pathways are involved in the complicated processes leading to bone regeneration.^89^, ^90^, ^91^, ^92^ Among all the cells and signalling pathways, type H vessel ECs have great capacity for coupling osteogenesis with angiogenesis during bone regeneration. At present, the lack of vascularization and functional blood vessels in tissue‐engineered bone has become the biggest obstacle to its clinical application.^93^, ^94^ Osterix^+^ osteoprogenitors are preferentially associated with type H capillaries in the metaphysis and endothelium due to the actions of various growth factors produced by ECs.^20^, ^23^ Therefore, administration of type H vessel inducers in bone tissue engineering, leading to an increase in type H vessels in the bone defect area, may be a promising therapeutic approach. Xu et al^17^ showed enhanced bone fracture healing and counteracted bone loss in a mouse model of postmenopausal osteoporosis by administration of recombinant SLIT3. Meanwhile, composite scaffolds incorporating bioactive lipids achieved efficient vascularized bone regeneration through synergistic induction of additional type H vessel formation, indicating coupling of angiogenesis and osteogenesis.^95^

However, there are some remaining controversies regarding studies of type H vessels. Deletion of the gene encoding integrin β1, a cell‐surface transmembrane glycoprotein, led to shorter femurs in mice, despite an increase in the number of type H vascular ECs. Furthermore, type H vessels that are not in an orderly alignment can eventually impair effective function in metabolism and decrease osteoprogenitor number and osteocalcin, thereby leading to a reduction in bone mass.^18^ Meanwhile, subchondral bone angiogenesis may play an important role in the pathogenesis of osteoarthritis.^96^, ^97^ In patients with osteoarthritis, changes in bone mass of the subchondral bone can accelerate remodelling of the subchondral bone, cause mechanical stress changes, accelerate joint cartilage degeneration, and eventually aggravate osteoarthritis.^98^, ^99^ Subchondral bone and articular cartilage comprise unified motion units that are integrated with one another and bear any biological damage load together.^100^ The subchondral bone is attached to the subchondral surface, providing mechanical support and cushioning to the cartilage.^101^, ^102^ During subchondral bone hyperplasia and remodelling, the bone mass is significantly increased with alterations in the intrabone microenvironment, and thus the changing mechanical load can aggravate osteoarthritis.^103^ Cui et al^88^ observed an accumulation of type H vessels around subchondral bone islands in osteoarthritis model mice. Lu et al found that type H vessels were increased in subchondral bone in osteoarthritis model mice and aged mice. In addition, mTORC1 activation in cartilage stimulated VEGF‐A production in articular chondrocytes and type H vessel formation in subchondral bone. Mechanistic target of rapamycin complex (mTORC) in chondrocytes promoted osteoarthritis partially through formation of VEGF‐A‐stimulated subchondral type H vessels.^104^ Moreover, type H EC‐derived Mmp‐9 has crucial roles in cartilage matrix invasion and promotion of bone formation.^105^ The above findings indicate that type H angiogenesis in subchondral bone may be an important feature of early osteoarthritis and suggest that the number of type H vessels can reflect the severity of subchondral bone hyperplasia and remodelling.

## 6 CONCLUSIONS

Type H blood vessels, a newly discovered blood vessel subtype mainly distributed in the epiphysis of bone, can promote an increase in bone mass and accelerate bone formation. These vessels also show strong positive staining with antibodies against two EC proteins (CD31 and EMCN). Because the number of type H vessels in aged individuals is much lower than that in young individuals, these vessels can serve as a sensitive marker for ageing and bone mass. Runx2^+^ and Osterix^+^ osteoprogenitors are densely distributed around type H vessels, thus confirming their role as a potent promoter for bone regeneration.

Type H vessels act as an important bone regeneration promoter at bone defect sites. On the one hand, the osteogenic progenitor cells surrounding type H vessels express the transcription factors Osterix and Runx2 at high levels to promote bone formation. On the other hand, type H vascular ECs secrete many factors, such as Noggin and VEGF, that stimulate the proliferation and differentiation of osteoprogenitors to regulate osteogenesis. Furthermore, type H vessels have a high tendency to differentiate into arteries that create the basis for a local vascular network in the bone defect area, which is crucial for bone regeneration. Several factors are involved in the coupling of type H vessel formation and osteogenesis. Administration of exogenous PDGF‐BB and activation of Hif‐1α and Notch signalling pathway in bone defect sites promote the formation of type H vessels, along with restoration of bone. In summary, type H vessels provide new insights for detection of the molecular and cellular mechanisms that underlie the crosstalk between angiogenesis and osteogenesis. As a result, more feasible therapeutic approaches for treatment of bone defects by targeting type H vessels may be applied in the future.

## CONFLICTS OF INTEREST

The authors declare no conflicts of interest.

## AUTHOR CONTRIBUTIONS

Jiankang Zhang contributed to manuscript writing and picture making; Jian Pan and Wei Jing contributed to paper design and revision.

## Data Availability

Data sharing is not applicable to this article as no new data were created or analysed in this study.
